# Principles of ADHD diagnosis in adults The diagnosis of ADHD in adults poses unique challenges due to the subtlety of symptoms and the presence of comorbidities

**DOI:** 10.1192/j.eurpsy.2024.128

**Published:** 2024-08-27

**Authors:** A. Todzia

**Affiliations:** Polish Psychiatric Association, Warsaw, Poland

## Abstract

**Abstract:**

Attention-Deficit/Hyperactivity Disorder (ADHD) is commonly associated with childhood, yet its prevalence and impact extend into adulthood. The diagnosis of ADHD in adults poses unique challenges due to the subtlety of symptoms and the presence of comorbidities.

The workshop aims to provide early career psychiatrists with a comprehensive understanding of the unique challenges associated with adult ADHD, emphasizing evidence-based approaches to diagnosis and effective management.

The management of adult ADHD involves a multimodal approach encompassing psychoeducation, pharmacotherapy, and psychosocial interventions. The integration of cognitive-behavioral therapy (CBT) and coaching strategies proves beneficial in addressing executive function deficits and enhancing adaptive skills.

Pharmacological interventions are a key component in the management of ADHD. These interventions aim to alleviate symptoms, improve cognitive functioning, and enhance overall functioning. The two main classes of medications commonly used for ADHD treatment are stimulants and non-stimulants.

**Disclosure of Interest:**

None Declared

